# A Multi-center Prospective Study for Implementation of an MRI-Only Prostate Treatment Planning Workflow

**DOI:** 10.3389/fonc.2019.00826

**Published:** 2019-08-29

**Authors:** Peter Greer, Jarad Martin, Mark Sidhom, Perry Hunter, Peter Pichler, Jae Hyuk Choi, Leah Best, Joanne Smart, Tony Young, Michael Jameson, Tess Afinidad, Chris Wratten, James Denham, Lois Holloway, Swetha Sridharan, Robba Rai, Gary Liney, Parnesh Raniga, Jason Dowling

**Affiliations:** ^1^Department of Radiation Oncology, Calvary Mater Newcastle, Newcastle, NSW, Australia; ^2^School of Mathematical and Physical Sciences, University of Newcastle, Newcastle, NSW, Australia; ^3^Liverpool Hospital Cancer Therapy Centre, South West Sydney Local Health District, Sydney, NSW, Australia; ^4^South Western Sydney Clinical School, University of New South Wales, Sydney, NSW, Australia; ^5^Hunter New England Imaging, HNE Health Service, Newcastle, NSW, Australia; ^6^School of Physics, University of Sydney, Sydney, NSW, Australia; ^7^Ingham Institute for Applied Medical Research, Sydney, NSW, Australia; ^8^Centre for Medical Radiation Physics, University of Wollongong, Wollongong, NSW, Australia; ^9^The Australian E-Health Research Centre, Commonwealth Scientific and Industrial Research Organisation, Brisbane, QLD, Australia

**Keywords:** MRI-only, synthetic CT, pseudo-CT, MRI-alone, prostate

## Abstract

**Purpose:** This project investigates the feasibility of implementation of MRI-only prostate planning in a prospective multi-center study.

**Method and Materials:** A two-phase implementation model was utilized where centers performed retrospective analysis of MRI-only plans for five patients followed by prospective MRI-only planning for subsequent patients. Feasibility was assessed if at least 23/25 patients recruited to phase 2 received MRI-only treatment workflow. Whole-pelvic MRI scans (T2 weighted, isotropic 1.6 mm voxel 3D sequence) were converted to pseudo-CT using an established atlas-based method. Dose plans were generated using MRI contoured anatomy with pseudo-CT for dose calculation. A conventional CT scan was acquired subsequent to MRI-only plan approval for quality assurance purposes (QA-CT). 3D Gamma evaluation was performed between pseudo-CT calculated plan dose and recalculation on QA-CT. Criteria was 2%, 2 mm criteria with 20% low dose threshold. Gold fiducial marker positions for image guidance were compared between pseudo-CT and QA-CT scan prior to treatment.

**Results:** All 25 patients recruited to phase 2 were treated using the MRI-only workflow. Isocenter dose differences between pseudo-CT and QA-CT were −0.04 ± 0.93% (mean ± SD). 3D Gamma dose comparison pass-rates were 99.7% ± 0.5% with mean gamma 0.22 ± 0.07. Results were similar for the two centers using two different scanners. All gamma comparisons exceeded the 90% pass-rate tolerance with a minimum gamma pass-rate of 98.0%. In all cases the gold fiducial markers were correctly identified on MRI and the distances of all seeds to centroid were within the tolerance of 1.0 mm of the distances on QA-CT (0.07 ± 0.41 mm), with a root-mean-square difference of 0.42 mm.

**Conclusion:** The results support the hypothesis that an MRI-only prostate workflow can be implemented safely and accurately with appropriate quality assurance methods.

## Introduction

The benefit of MRI scanning for prostate radiation therapy planning is-well established with studies demonstrating lower inter-observer variation in contours, and smaller contours than CT with subsequent lower doses to normal tissues such as the penile bulb (1–3). The use of MRI for prostate delineation therefore potentially allows for more accurate and more consistent treatment. Typically for prostate planning as well as other treatment sites the MRI scans are registered to CT scans to allow for dose computation using the electron density or physical density map that is generated from simple calibration of Hounsfield Units (HU). The registration can be performed using MRI sequences that visualize implanted gold fiducial markers or less accurately using prostate soft-tissue. The MRI scans are often not acquired in the treatment position and do not encompass the patient external contour. The major limitation of this approach is that systematic registration uncertainties can result in the prostate contour from MRI (the target which is used to generate the high dose region) being misaligned to the gold fiducial positions on CT which are used for image guidance. These uncertainties have been estimated to be up to 2 mm in standard deviation which are significant given the small margins used for modern high dose treatments. These are systematic targeting uncertainties present for every treatment fraction.

Recently a new paradigm for treatment planning has emerged that of MRI-alone or MRI-only planning (4–13). In this approach the HU map for dose calculation is generated from one or more MRI sequences that encompass the typical field of view of a planning CT scan (pseudo/synthetic CT). A variety of methods have been developed to convert MRI data to HU including calibration and classification methods using the MRI voxel values, atlas-based methods that use deformable image registration, hybrid voxel, and atlas methods and deep-learning algorithms (convolutional neural networks and generative adversarial networks) (14, 15). The performance of these algorithms are similar and meet the requirements for dose calculation accuracy. Clinical acceptance is assessed by comparison of dose calculation on CT and pseudo-CT for individual patients. The increase in dose calculation uncertainty is regarded as a worthwhile trade-off to eliminate the systematic registration uncertainty (4).

While there have been many investigations performed retrospectively comparing new pseudo-CT methods to CT dose calculations, there has been less attention to clinical implementation of MRI-only workflows and in particular how these can be performed and assessed to ensure safe clinical use. Tyagi et al. presented a clinical workflow for MRI-only simulation (16). Their workflow included an initial CT simulation appointment where orthogonal x-ray scout images were used to determine patient dimensions and acceptance for use by the commercial MRCAT synthetic CT software. If the patient had prior brachytherapy a small field-of-view CT scan was acquired to distinguish brachytherapy seeds from fiducial markers. Forty-two patients from an initial cohort of 48 received this workflow. Tenhunen et al. presented their experience with MRI-only prostate planning for a large cohort at Helsinki hospital (17). They found that 92% of patients were suitable for MRI-only workflow. To date these reports are for single institution studies.

MRI-only treatment planning is an entirely new approach for treatment centers and does entail potential risks. Recently a failure modes and effects analysis (FMEA) of MRI-only treatment planning was reported which demonstrated multiple failure modes that need to be considered (18). To gain benefit from these techniques it is important that MRI-only workflows be implemented in a rigorous and safe manner with appropriate quality assurance methods. In this work a multi-center study was initiated for the implementation of an MRI-only prostate workflow. Two different treatment centers participated and 30 patients in total were recruited, 15 at each center. The study was designed to enable and assess safe implementation of this new technique for radiation therapy departments.

## Methods and Materials

### Patients

Thirty patients receiving radical radiation oncology treatment for prostate cancer were recruited across two treatment centers. The study title was High precision Prostate Substitute CT based External beam Radiotherapy (HIPSTER). The study was ethically approved by the Hunter New England Human Research Ethics Committee (HREC Registration No: 16/07/20/3.01, NSW HREC Reference No: HREC/16/HNE/298, Australian New Zealand Clinical Trials Registry ACTRN12616001653459) and informed consent was obtained from all patients. The study opened for recruitment 6 April 2017 and closed to recruitment 16 April 2019 with 15 patients recruited at each center. Eligibility criteria were men >18 years, low, intermediate or high risk prostate cancer, fiducial gold markers inserted and prostate or prostate and seminal vesicle irradiation. The exclusion criteria were inability to undergo MRI scanning, prior pelvic radiation therapy, unsafe for or refusal to undergo fiducial marker insertion, presence of hip prostheses, men highly dependent on medical care or men with mental or intellectual impairment that would have difficulty giving informed consent to the study. Patient details are listed in Table 1. Three fiducial markers were implanted at least 1 week prior to MRI scanning. Treatment details are listed in Table 2. Patients were scanned and treated according to local guidelines except for the MRI-only planning requirements outlined below.

**Table 1:** Patient details.

**Patient detail**	**Mean [range]**
Age (years)	73.4 [58–83]
Gleason score	3 + 3 = 6 (*n* = 3), 3 + 4 = 7 (*n* = 13), 4 + 3 = 7 (*n* = 10), 4 + 4 = 8 (*n* = 1), 4 + 5 = 9 (*n* = 3)
Pre-treatment PSA	9.0 [0.88–33.8]
Weight (kg)	84.4 [62–122]
Body mass index (BMI)	28.5 [19–39]

**Table 2:** Details of the centers equipment and techniques.

	**Center 1**	**Center 2**
CT scanner	Toshiba Acquilion or GE LightSpeed-RT or Siemens Confidence	Philips Brilliance Big Bore
MRI scanner	Siemens Magnetom Skyra 3T	Siemens Magnetom Skyra 3T
Treatment planning system	Varian Eclipse	Philips Pinnacle
Record and verify system	Varian Aria	Elekta Mosaiq
Linear accelerators	Varian Clinac or Truebeam	Elekta Synergy (Agility MLC) or Versa
Fiducial markers	1.0 × 3.0 mm gold	1.2 × 3.0 mm gold
Treatment technique	7-field IMRT (*n* = 11) 2-arc VMAT (*n* = 4)	1-arc VMAT (*n* = 12) 2-arc VMAT (*n* = 3)
Prescribed dose	60 Gy in 20 fractions (*n* = 14) 78 Gy in 39 fractions (*n* = 1)	60 Gy in 20 fractions (*n* = 9) 78 Gy in 39 fractions (*n* = 6)
Beam energy	6 MV (*n* = 13) 10 MV (*n* = 2)	6 MV

### Centers and Equipment

While the centers had the same make and model of 3T MRI scanner (MAGNETOM Skyra, Siemens Healthineers, Erlangen, Germany) they differed in all other radiation therapy equipment. Both MRI scanners were fully equipped as MRI simulators with radiation therapy flat couch tops (CIVCO Medical Solutions, Coralville, USA), laser bridges (LAP Laser, Luneburg, Germany) and pelvic coil bridges (CIVCO). Both scanners had regular quality assurance procedures for image quality and distortion.

### Study Design

**Figure 1:**
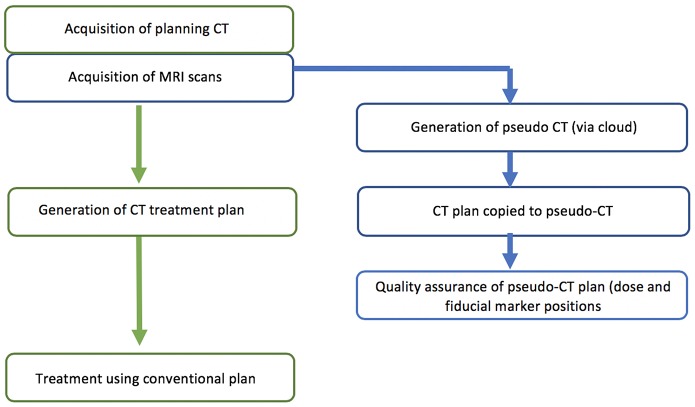
Phase 1 design for retrospective analysis.

**Figure 2:**
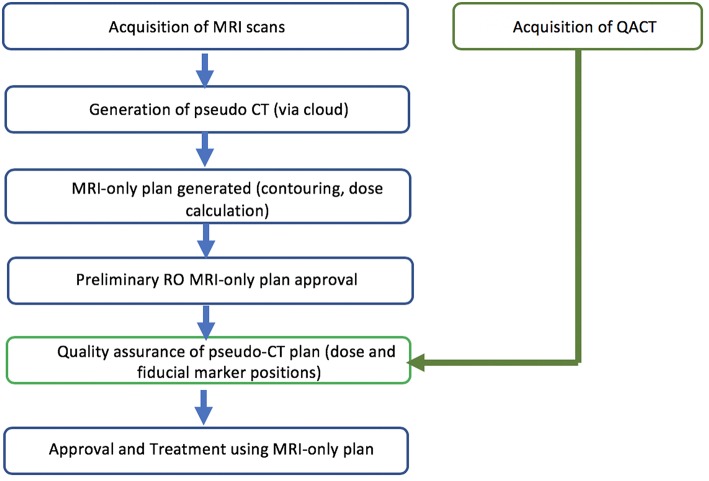
Phase 2 study design for prospective MRI-only planning.

The study was designed as a two-phase implementation model where centers performed retrospective analysis of MRI-only plans for five patients followed by prospective MRI-only planning for subsequent patients. The first phase is commensurate with literature studies to determine the accuracy of the pseudo-CT generation (Figure 1). The second phase is designed as a transition to MRI-only planning without CT where the MRI-only workflow is implemented but with final quality assurance to ensure accuracy and safety provided by comparison to CT scanning. In this phase the CT scan (QA-CT) is only imported into the TPS following preliminary radiation oncologist approval of the MRI-only plan (Figure 2). The study aimed to recruit and treat 25 patients with phase 2 MRI-only prospective planning. As center 1 had previously performed retrospective analysis for 39 patients (19) they began at phase 2. Center 1 recruited 15 patients to phase 2 while center 2 recruited five patients to phase 1 and a further 10 patients to phase 2.

The major endpoint of the study was feasibility of MRI-only implementation with the aim achieved if >90% of patients received MRI-only treatment. This allowed for 2/25 patients to have their MRI-only plans deemed unacceptable. From previous experience 39/39 patients would have achieved the dose calculation criteria therefore a 25 patient sample size was regarded as reasonable to recruit and to demonstrate feasibility. Secondary endpoints were the assessment of the dose and image-guidance quality assurance metrics.

### MRI Simulation

**Figure 3:**
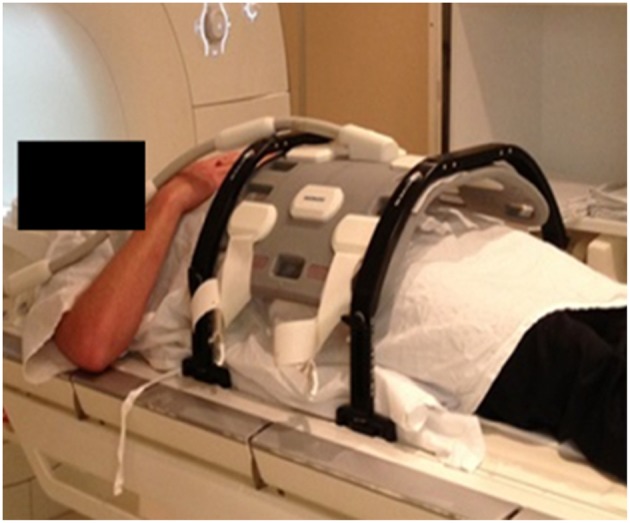
Example patient setup for MRI simulation.

The patients were setup in exactly the same position as for treatment with a radiation therapist in attendance for patient setup. Patients were aligned using the lasers and MRI visible skin markers (Liquimark, Suremark) were placed on the patient's skin along with temporary tattoo marks. The coil mount was placed over the patient's pelvis, without compressing their contour. All patients were positioned head-first supine and had full bladder and empty rectum. An example of patient positioning for MRI simulation is shown in Figure 3.

A large field of view 3D sequence was utilized for pseudo-CT generation. Both centers used the same T2-weighted SPACE isotropic 3D sequence with 1.6 mm voxel side dimensions and scanning parameters as previously reported (19). The manufacturers 3D distortion correction was used for all scans.

Routine sequences used at each center were also acquired for prostate contouring and fiducial marker visualization. These were not altered for this study as the aim was to follow the conventional workflow as closely as possible but with MRI replacing the functionality of CT for treatment dose calculation, contouring and image-guidance. Details of these sequences have been reported earlier (19). The functionality of the three main sequences acquired along with the pseudo-CT are shown in Table 3. A checklist was designed to ensure adequate MRI scanning for treatment planning shown in Table 4.

**Table 3:** Details of the MRI scans acquired and their function for MRI-only planning.

**Scan type**	**Function**
Small field-of view T2 TSE	Prostate delineation (CTV) and urethral delineation
Small field-of-view T1 GRE Flip 80	Fiducial marker delineation
Large field-of-view T2 SPACE	Organ delineation Generation of pseudo- CT
Pseudo-CT	Dose calculation Image guidance using fiducial marker contours transferred from T1 flip 80

**Table 4:** MRI simulation checklist.

MRI skin markers placed on tattoos, patient level with lasers (scanned HFS)	□
LFOV MRI acquired first	□
LFOV MRI—skin markers visible on LFOV MRI and patient is leveled (within 0.5 cm)	□
LFOV MRI—covers external body contour in all directions and inferior superior extent according to CT scanning guidelines	□
LFOV MRI—T2 SPACE, 1.6 mm isotropic voxels	□
LFOV MRI—3D distortion correction is active	□
SFOV T2 TSE—3D distortion correction is active	□
SFOV T1 GRE flip80—three gold markers are visible on the scan, 2D distortion correction is active	□

### Pseudo-CT Generation

Details of the pseudo-CT method have been reported in detail previously (19). The method is a hybrid atlas-voxel method using an atlas of 39 previously acquired patients. The LFOV SPACE sequence was de-identified and the patient details replaced with a study ID before cloud upload to a secure site. The pseudo-CT was generated and downloaded to the treatment center where the patient ID and details were entered into the DICOM header of the scan, replacing the study ID. For the first eight patients the pseudo-CT generation was identical to the method described in Dowling et al. (19) including the addition of an extra 1.0 mm “skin” expansion due to the lack of visibility of this layer on MRI. However, this was discontinued due to erroneous generation of this layer for patient number 9 of the study and it was decided that it was clinically more robust to subsequently exclude this additional layer calculation from the algorithm.

### CT Scanning

All patients received CT scans (QA-CT) for quality assurance and analysis of the MRI-only workflow performed as close as possible in time as the MRI scan and preferably after the MRI scan. Slice thickness was 2.0 mm or 2.5 mm at Center 1 and 2.0 mm at Center 2.

### MRI-Only Treatment Planning

The MRI sequences along with the pseudo-CT were imported into the treatment planning system (TPS). Alignment of all scans was visually checked by a radiation therapist. Following prostate, organ and fiducial marker delineation these contours were transferred to the pseudo-CT for treatment plan generation following incorporation of a couch-model. Imbedding of fiducials into the pseudo-CT scan pixel values was not used. Treatment plans were then defined according to routine department protocols. The pseudo-CT with attached fiducial marker contours was then transferred to the linear accelerator for image guidance with either cone-beam CT based image registration to pseudo-CT based on the markers or orthogonal kilovoltage x-ray image based image registration to digitally reconstructed radiographs generated from pseudo-CT.

### Quality Assurance

A quality assurance procedure was designed for assessment of MRI-only treatment plans prior to acceptance of the plan for treatment. This included verification that the scans were consistent, pseudo-CT appearance and field-of-view, seeds were correctly identified, and dose and image-guidance metrics as described below (Table 5). This procedure is designed for an implementation phase for MRI-only planning where a MRI-only workflow is used but a gold-standard CT scan is still acquired for final verification before MRI-only plan is used for treatment.

**Table 5:** Quality assurance checklist for MRI-only plan.

Distortion correction	Confirm that 3D distortion correction was activated for the whole-pelvic scan. Check distortion corrections for other scans.	□
Image transfer	Confirm that pseudo-CT corresponds to the MRI scan and conventional CT scan to verify that correct pseudo-CT has been assigned to the patient.	□
Image orientation and appearance	Confirm that pseudo-CT is correctly oriented by comparison to conventional CT scan. Visually inspect the entire pseudo-CT volume and compare to conventional CT for any missing tissue or major differences.	□
Field of view	Ensure that the pseudo-CT has sufficient field-of-view to cover all external contours and sufficient extension superiorly and inferiorly for dose calculations.	□
Fiducial marker visibility	Verify that the fiducial marker structures generated on the pseudo-CT correspond to the fiducial markers determined from the conventional CT (i.e., all fiducial marker locations have been correctly identified).	□
Femoral heads	Confirm visually that MRI generated bone contours visually correspond to CT bone contours.	□
Dose at isocenter	Verify that isocenter dose on pseudo-CT is within 2% of conventional CT	□
Dose distribution	Verify that 3D Gamma comparison at 2%, 2 mm criteria > 90% pass-rate for the entire body volume (−1.5 cm to avoid skin region where dose is uncertain).	□
Fiducial marker positions	Verify that fiducial marker contours on pseudo-CT are within 1 mm from centroid of the locations on conventional CT from centroid (accounting for prostate rotation).	□

Following full preparation of the MRI-only treatment plan and preliminary radiation oncologist approval, the QA-CT scan was imported into the TPS. This scan was registered to the pseudo-CT using automatic registration and the MRI plan transferred to the QA-CT. Dose was recalculated on the QA-CT using the same fluences and monitor units as the MRI plan. Following alignment using the isocenters the doses were interpolated onto a 1.5 mm voxel size and compared with a three-dimensional gamma calculation. A 20 mm region close to the skin was excluded from the comparison using a two-dimensional erosion operation on each axial plane to avoid the large dose discrepancies due to differences in the external body contour at CT and MRI. A dose threshold of 20% of the maximum dose was used and gamma criteria of 3%, 3 mm, 2%, 2 mm, and 2%, 1 mm with the QA-CT as the reference dose for the comparison. Doses at the isocenter were also compared. Acceptance criteria for the dose calculation on pseudo-CT were isocenter dose within 2% and gamma pass-rate > 90% at 2%, 2 mm criteria.

Locations of fiducial markers as identified on MRI were also compared to locations on the QA-CT scan. The x, y, and z locations of the markers were carefully measured on the scans and entered into an Excel spreadsheet which calculated the centroid of the markers for each scan. The distances of each marker to the centroid were calculated and compared for the scans. If all distances were within 1.0 mm then the MRI locations were accepted.

## Results

**Figure 4:**
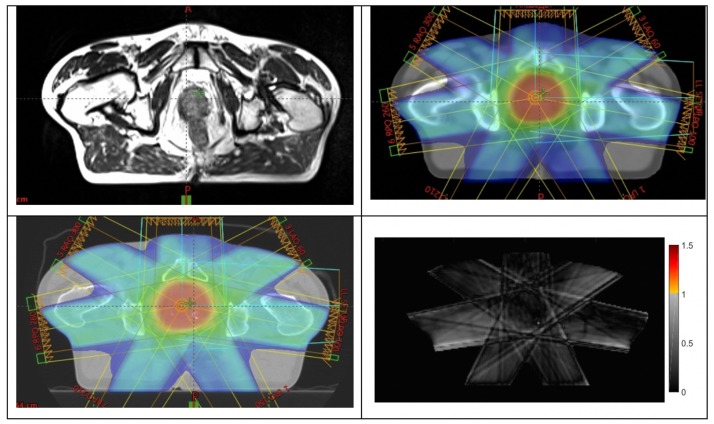
Example of a patient (top-left) large-field-of-view MRI scan; (top-right) dose plan developed on pseudo-CT; (bottom-left) dose recalculated on QA-CT scan; (bottom-right) gamma analysis result at 2%, 2 mm criteria.

The primary outcome of the study was achieved with all 25 patients in phase 2 having their MRI-only plans accepted by the radiation oncologist and passing all quality assurance criteria. These patients were all treated using the MRI-only workflow. Figure 4 shows an example patient MRI scan, pseudo-CT dose calculation, and QA-CT dose recalculated for comparison.

**Figure 5:**
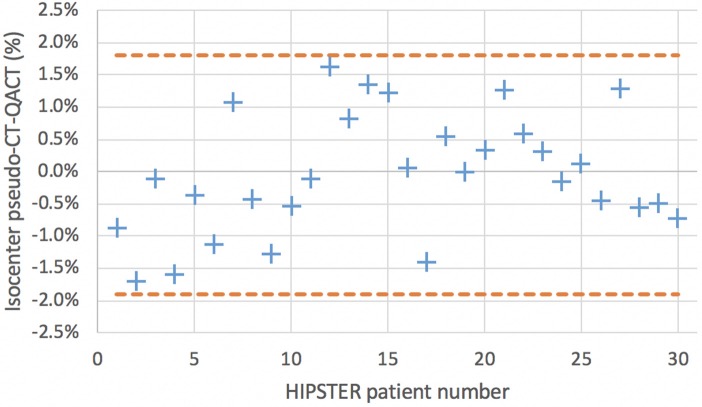
Isocenter dose comparison on MRI only pseudo-CT scan and QA-CT scan.

For the secondary endpoints all 30 patient results were assessed including the five patients for phase 1 at center 2 as the assessment methodology is the same as phase 2. The results for the ratio of isocenter dose on pseudo-CT and QA-CT are shown in Figure 5 along with the Bland-Altman levels. The mean difference in isocenter doses was −0.04% with a standard deviation of 0.93%. The effect of the first eight patients calculated with the 1.0 mm skin expansion can be seen with lower pseudo-CT doses. The mean difference for the first eight patients was −0.64% (0.90%) while for the subsequent patients it was 0.17% (0.85%). All isocenter dose differences were within 2.0% and only 3 (10%) had more than 1.5% difference.

The results for the gamma evaluations of the dose on pseudo-CT and QA-CT are shown in Table 6 for the three gamma criteria. The average gamma pass-rates and the average of the mean gamma values for all patients are shown.

**Table 6:** Results of gamma analysis for comparison of dose calculation using pseudo-CT and CT.

	**3%, 3 mm**	**2%, 2 mm**	**2%, 1 mm**
Gamma pass-rate (%)	100.0	99.7	99.2
Standard deviation (%)	0.1	0.5	1.0
Mean gamma	0.145	0.218	0.221
Standard deviation	0.05	0.07	0.07

**Figure 6:**
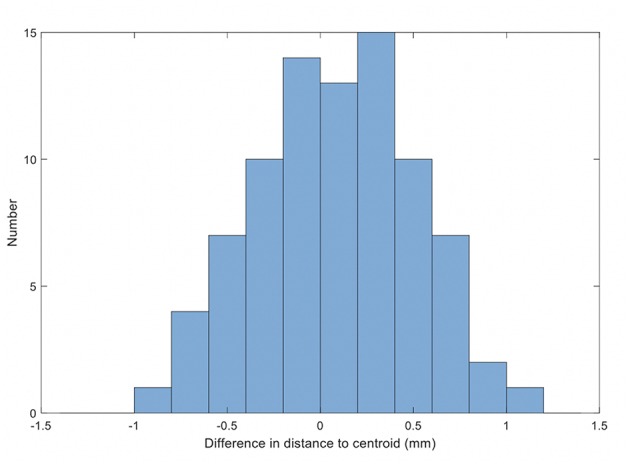
Histogram of the differences in distance on MRI and QA-CT of each marker to the centroid of the markers.

The results for the comparison of fiducial marker distances to centroid on MRI and QA-CT are shown in Figure 6. The average difference between MRI and QA-CT was 0.07 mm (1 SD = 0.41 mm) and the root-mean-square difference was 0.42 mm. The maximum difference was 1.00 mm.

## Discussion

This study demonstrates that MRI-only workflows can be implemented in a multi-center setting with appropriate quality assurance measures to ensure accurate and safe treatment. The study is distinct from most other reported studies in that it is prospective and the 25 patients received an MRI-only workflow and treatment.

The QA-CT scan was only used for quality assurance purposes and this was imported following full generation and preliminary approval of the MRI only plan. This ensures that an MRI-only planning workflow is fully implemented but also allows for verification against the gold standard for dose calculation and image-guidance. This prepares the center for MRI-only workflow and ensures safe practice. Subsequent to this implementation phase the center could then use the MRI-only workflow without CT acquisition. There are two major potential approaches to this; consider that there is now adequate confidence in the process that no specific quality assurance techniques are required; or to utilize separate quality assurance techniques, and these decisions will be center-dependent. To provide a method for the latter approach, in parallel with this study a simple bulk-density calculation method was developed to compare to the pseudo-CT dose calculation. This was based on MRI bone and body contour anatomy and the results will be reported separately. This method can provide confidence in the integrity of the pseudo-CT and is robust and easy to perform.

Quality assurance methods to validate fiducial marker positions as identified on MRI scans would also be beneficial. Prostate calcifications can in some cases be difficult to differentiate from fiducial markers in the sequences used here. Although potentially problematic this misidentification is unlikely to lead to image guidance errors as the seed positions are clearly identified with cone-beam CT scans or x-ray images prior to treatment and misidentified seed positions are obvious and can be corrected. However, this is not an ideal scenario as it could delay treatment. Several methods have been proposed to ensure fiducial marker identification using MRI techniques or planar x-ray imaging (16, 20–25).

Patient movement between and during MRI scans is also a potential source of error in MRI-only planning as the scans can take several minutes to acquire. It is critical to ensure that movement has not occurred between the small field-of-view acquisitions used for CTV definition and fiducial marker delineation. If there is a shift of position between them this will introduce a systematic error in dose delivery to the CTV/PTV. Visual inspection of alignment of the prostate contour on the two sequences should be performed. Note that this is not a problem specific to MRI-only planning. This problem also exists for MRI-CT registration based treatment planning as is currently performed. Movement of the patient for the large-field-of-view MRI scan is not as critical for dose calculation but it will result in systematic errors of normal tissues that are delineated on this scan and hence potential mismatch of planned and delivered doses to these organs.

For patient 9 an error in the pseudo-CT scan was detected visually during plan generation. This was due to the algorithm component that introduces an additional “skin” expansion to compensate for the lack of visibility of the skin on MRI. Although this correction was introduced in earlier method development to improve dose calculation accuracy it was felt that it would be clinically safer to exclude this additional layer for this and subsequent patients. This patients pseudo-CT was recalculated with the modified algorithm which generated a new pseudo-CT that was used for the treatment plan. This has a small effect on the dose calculation when compared to QA-CT. The patients prior to patient 9 that included this layer had on average slightly lower dose calculation on pseudo-CT compared to CT whereas the patients subsequent to the change had on average slightly higher dose on pseudo-CT when compared to CT. The patients prior to the change could be recalculated with the modified algorithm however the patient plans were developed using the prior algorithm so this would not reflect the reality for this prospective study.

## Conclusion

An MRI-only workflow was introduced in a prospective multi-center trial setting and all recruited (25 patients) received the MRI-only workflow. MRI-only planning workflow can be implemented in a safe manner with appropriate testing and quality assurance.

## Data Availability

The datasets generated for this study are available on request to the corresponding author.

## Author Contributions

PG: principal investigator, project manager, and wrote manuscript. JM and MS: patient recruitment, treatment planning, and clinical guidance. PH and PP: patient data acquisition, treatment planning, and protocol development. JC: data analysis and reporting of results. LB and GL: patient data acquisition and imaging development. JS: patient recruitment, data management, and ethics management. TY: patient data acquisition, data analysis, and quality assurance. MJ and LH: patient data acquisition, data analysis, and protocol development. TA: patient recruitment, data management, and quality assurance. CW and JDe: patient recruitment and clinical guidance. SS: patient recruitment, clinical guidance, and procedures development. RR: patient data acquisition, imaging development, and protocol development. PR and JDo: synthetic CT generation and technical development.

### Conflict of Interest Statement

The authors declare that the research was conducted in the absence of any commercial or financial relationships that could be construed as a potential conflict of interest.
